# Morphological Fabrication of Rubber Cutaneous Receptors Embedded in a Stretchable Skin-Mimicking Human Tissue by the Utilization of Hybrid Fluid

**DOI:** 10.3390/s21206834

**Published:** 2021-10-14

**Authors:** Kunio Shimada, Ryo Ikeda, Hiroshige Kikura, Hideharu Takahashi

**Affiliations:** 1 Faculty of Symbiotic Systems Sciences, Fukushima University, 1 Kanayagawa, Fukushima 960-1296, Japan; 2 Institute of Innovative Research, Tokyo Institute of Technology, 2-12-1 Ookayama, Meguro-ku, Tokyo 152-8550, Japan; ikeda.r.ah@m.titech.ac.jp (R.I.); kikura@lane.iir.titech.ac.jp (H.K.); htakahashi@lane.iir.titech.ac.jp (H.T.)

**Keywords:** sensor, cutaneous receptors, skin, mimesis, rubber, electrolytic polymerization, hybrid fluid (HF), magnetic compound fluid (MCF), humanoid, soft robotics

## Abstract

Sensors are essential in the haptic technology of soft robotics, which includes the technology of humanoids. Haptic sensors can be simulated by the mimetic organ of perceptual cells in the human body. However, there has been little research on the morphological fabrication of cutaneous receptors embedded in a human skin tissue utilizing artificial materials. In the present study, we fabricated artificial, cell-like cutaneous receptors embedded in skin tissue mimicking human skin structure by utilizing rubber. We addressed the fabrication of five cutaneous receptors (free nerve endings, Krause and bulbs, Meissner corpuscles, Pacinian corpuscles and Ruffini endings). In addition, we investigated the effectiveness of the fabricated tissue for mechanical and thermal sensing. At first, in the production of integrated artificial skin tissue, we proposed a novel magnetic, responsive, intelligent, hybrid fluid (HF), which is suitable for developing the hybrid rubber skin. Secondly, we presented the fabrication by utilizing not only the HF rubber but our previously proposed rubber vulcanization and adhesion techniques with electrolytic polymerization. Thirdly, we conducted a mechanical and thermal sensing touch experiment with the finger. As a result, it demonstrated that intelligence as a mechanoreceptor or thermoreceptor depends on its fabric: the HF rubber sensor mimicked Krause and bulbs has the thermal and pressing sensibility, and the one mimicked Ruffini endings the shearing sensibility.

## 1. Introduction

Sensors are required in both hard and soft robotics [1]. Hard robotics deals with rigid structural materials actuated by mechatronic definitions with powered controllers and computers, while soft robotics predominantly utilizes soft materials involving elastomers, thin, flexible sheets, liquids, etc. Rubber is suitable as a soft material because it is highly elastic and compressible. Polydimethylsiloxane (PDMS), for example, is currently much used as a silicone rubber (Q) [2]. Q is also tractable and is a major commercial material in molding. Many studies have been carried out and many propositions have been made regarding functionalizing sensory systems in robotics. In hard robotics, sensors are ordinarily installed in or on the materials. In contrast, in soft robotics, variegated sensors with electrodes [3], such as Hall Effect sensors [4], pressure sensors [5], etc., are embedded in the soft material [6].

However, material that has sensors installed or embedded becomes fragile due to large tensile or compressive motion in both hard and soft robotics. In addition, in the case of soft robotics, sensing intelligence is often provided in the soft material, which utilizes rubber that is conductive due to a compounding filler such as carbon black [7] or magnetic powder [2]. The addition of such a filler into a material such as Q makes the material mechanically harder.

We can propose biological mimicry as another way to resolve the above inconvenience for both wide and soft motion and for both hard and soft robotics. Sensors are indispensable to obtain information about force, temperature, and so on, and a robot particularly requires the sensors’ haptic intelligence and structured sensing systems. Current robots either have an outer body of rigid plastic or have no outer body to cover the bare machinery, with the result that the robot would not necessarily be familiar with human society. Therefore, it is desirable to be able to embed sensors in an outer body that envelopes the machinery. The use of tactile rubber with sensibility fulfills this prerequisite because this type of rubber also has large elasticity and compressibility, like human skin [8].

Regarding biological mimicry, we can utilize a mimetic organ of perceptual cells in the human body. Perceptual cells are categorized into several types based on configuration and sensory intelligence. Configuration is based on the shape of the nerve endings of the receptor, for example, Meissner corpuscles, Pacinian corpuscles, Merkel cells, Ruffini endings, etc., and receptors include mechanoreceptors, thermoreceptors, etc. Until recently, they have been investigated with an eye to simulating them by replicating or mimicking their intelligence using mechanical or electronic devices with the application of stimuli [9,10,11]. The devices are not embedded in artificial skin such as rubber, but rather are externally structured gadgets with machinery and electric circuits to demonstrate mechanoreception or thermoreception. In addition, for the purpose of creating a humanoid with skin inspired by stimuli, electrical circuits with electrodes that are flexible and stretchable to deal with mechanical stress/strain have been utilized in thin film [12,13]; this corresponds to wearable devices installed on a human body involving the arm or hand. Although the demonstrated mechanoreceptors and thermoreceptors have been simulated as haptic sensors, they have been so large that they have not been a cell-like fabric in a human body at a μm-scale. To date, there have not been studies on the morphological fabrication of cutaneous receptors and human skin tissue using artificial materials. Therefore, the production of artificial skin embedded with artificial cutaneous receptors utilizing a rubber is a fascinating and invaluable subject.

Therefore, in the present study, we fabricated artificial cutaneous receptors embedded in artificial rubber skin mimicking human skin tissue. We propose a fabrication technique using rubber involving magnetic responsive fluid for cutaneous receptors mimicking human perceptual cells and a body part, the finger, with the rubber sensor embedded in urethane rubber (U). In general, the issues of the mixture of any rubbers and magnetic responsive fluid have not been conducted until recent except for our serial investigations, because the physical mechanism is elucidated and complicated.

However, the issues are significant for the development of the production of magnetic rubber. Therefore, the mixture of U-rubber and magnetic responsive fluid such as the present study is invaluable. Our proposed fabrication technique also includes the utilization of our previously proposed technique of adhesion of electric wires onto the rubber sensor and the rubber for the purpose of avoiding electric wires’ desorption from the sensor by large elongation or deformation of the body. The key issue of the present paper is the preliminary work of trying the fabrication of the mimic organ. Then, as a first step, we presented the viability of the fabrication of a finger involving simulated cutaneous receptors for both mechanical and thermal sensing: forcing, thermo and shearing sensibility, which are demonstrated as typical cases in our daily life: touching a body, touching a hot or cold water in washing a face or bathing, or robbing a rough body surface.

## 2. Hybrid Fluid (HF)

In order to take advantage of the defense against deterioration of non-diene rubber, diene and non-diene rubbers are combined using polyvinyl alcohol (PVA) [14]. Diene rubbers such as natural rubber (NR) and chloroprene rubber (CR) are soluble in water, while non-diene rubbers such as Q are soluble in kerosene. In particular, Q is soluble in silicone oil with PDMS. Therefore, rubber latex combined with NR (Ulacol; Rejitex Co., Ltd., Atsugi, Japan), CR (671A; Showa Denko Co., Ltd., Tokyo, Japan) and Q can be soluble in water, kerosene or silicone oil. For the possible combination of rubber and magnetic responsive fluid such as magnetic fluid (MF), the magnetic responsive fluid may include water, kerosene, or silicone oil. Where MF is both expensive and inconvenient as it must be purchased from a company, it cannot be easily produced in laboratory and its constituents are obscure because of a confidential engineering patent. Therefore, there is a new magnetic responsive fluid that replaces MF consists of magnetic particles and surfactant, which is simulated MF whose Fe_3_O_4_ is coated by a surfactant.

In the present study, we propose a cutting-edge magnetic responsive fluid, HF (hybrid fluid). HF consists of Fe_3_O_4_ (Fujifilm Wako Chemicals Co., Ltd., Osaka, Japan) and Fe (M300, about 50-μm particles; Kyowa Pure Chemical Co., Ltd., Tokyo, Japan) particles in the solution mixed with water, kerosene, or silicone oil (KF96 with 1-cSt viscosity with PDMS; Shin-Etsu Chemical Co., Ltd., Tokyo, Japan) with a surfactant of sodium hexadecyl sulfate aqueous solution (Fujifilm Wako Chemicals Co. Ltd., Osaka, Japan). Where KF96 contains PDMS, M300 should be compounded because the mixed fluid has little response to a magnetic field when we used only Fe_3_O_4_. The purpose of using the surfactant is to optimize the combination of diene and non-diene rubbers, as shown in our previous study [15]. HF can be produced as follows. First, sodium hexadecyl sulfate is combined with water at a weight ratio of 1:10. This sodium hexadecyl sulfate aqueous solution was compounded with Fe_3_O_4_ and M300 using an agitator. Next, the mixture was agitated for about 1 h with water, kerosene, KF96 and PVA. The use of a high-speed agitator or the mixing under vacuum optimizes dispersion enough to avoid the separated state of the mixed liquids. We should pay attention of degassing from the liquids since the liquids inflate by the agitating. If we do not use the vacuum pump during agitating, the liquids must be vacuumed intermittently in the interval of agitating. The constituents of HF are 3 g water, 3 g kerosene, 3 g KF96 (1cSt), 21 g PVA, 3 g Fe_3_O_4_, 3 g M300, 4 g sodium hexadecyl sulfate aqueous solution, and they are optimum in terms of miscibility with Q, NR, and CR.

When a magnetic field is applied, many needle-like magnetic clusters are created, such as our proposed magnetic compound fluid (MCF) as one of magnetic responsive fluid that is tackled in the issues of the mixture of any rubbers and magnetic responsive fluid. MCF is an intelligent colloidal fluid that is responsive to a magnetic field and is constructed by 1-μm ordered metal particles such as Ni, Fe, Cu, etc. and 10-nm ordered sphere magnetite (Fe_3_O_4_) particles in a solvent such as water, kerosene, silicone oil, etc., since MCF is made by compounding metal particles and MF. Regarding the combination of metal particles into a rubber, there have been many investigations on the combination of particles such as carbon, metal, etc. in the rubbers of NR [16], CR [17], nitrile rubber (NBR) [18], styrene-butadiene rubber (SBR) [19], Q [20,21], and on the combination of NR and SBR [22,23,24,25], NR and NBR [25], NR and ethylene-propylene rubber (EPM or EPDM) [26]. However, there are few reports of combining magnetic responsive fluid into a rubber except for the rubber involved MCF or HF of our serial investigation.

**Figure 1:**
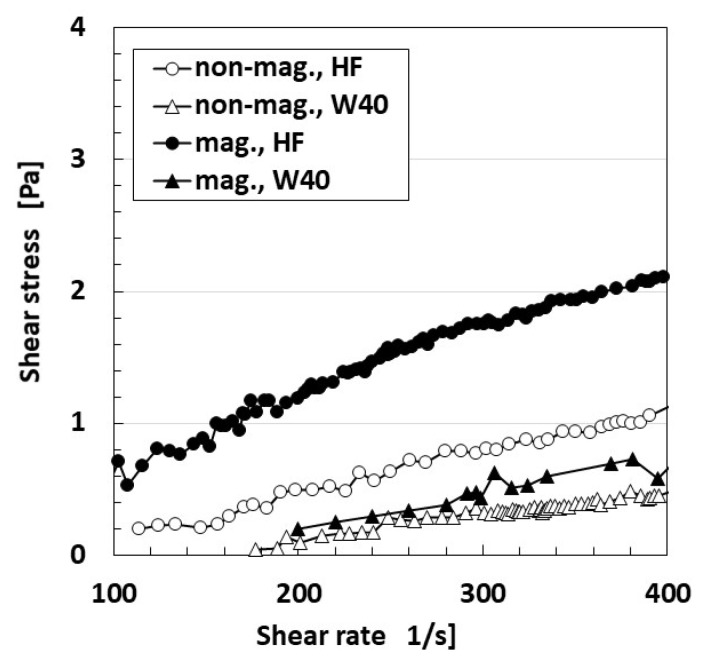
Relationship between shear rate and shear stress of HFs both without a magnetic field (non-mag.) and under a magnetic field of 39 mT (mag.).

The relationship between shear rate and shear stress is shown in Figure 1. W40 in the figure indicates a water-based MF with 40 wt% Fe_3_O_4_ (W40; Taiho Industry Co., Ltd., Takatsuki, Japan), for comparison with the HF, which replaces MF. For measurement, we used a plate-plate type rotational viscometer in order to achieve uniform distribution of the magnetic field over the rotational plate with a 40-mm diameter throughout. Torque applied on the plate was measured by torque meter (SS-005, Ono Sokki, Co. Ltd., Yokohama, Japan) under a rotational speed of the plate applied by DC motor (Vexta, BXM460-GFH2, Oriental Motor, Co. Ltd., Tokyo, Japan). The distance between the plates was 1 mm. 39-mT magnetic field was applied by a permanent magnet. Given this result, when no magnetic field was used, HF has almost the same quantitative ordered viscosity of MF, which also has the same ordered viscosity (water, kerosene, KF96). Under the application of a magnetic field, the viscosity of HF is multiplied several times, which is the same tendency seen with MF. However, the viscosity does not become greater than that of other magnetic responsive fluids, such as MRF. In general, the fluid polymerized by emulsifier has a comparatively low viscosity. HF is guessed to have a similar peculiarity.

**Figure 2:**
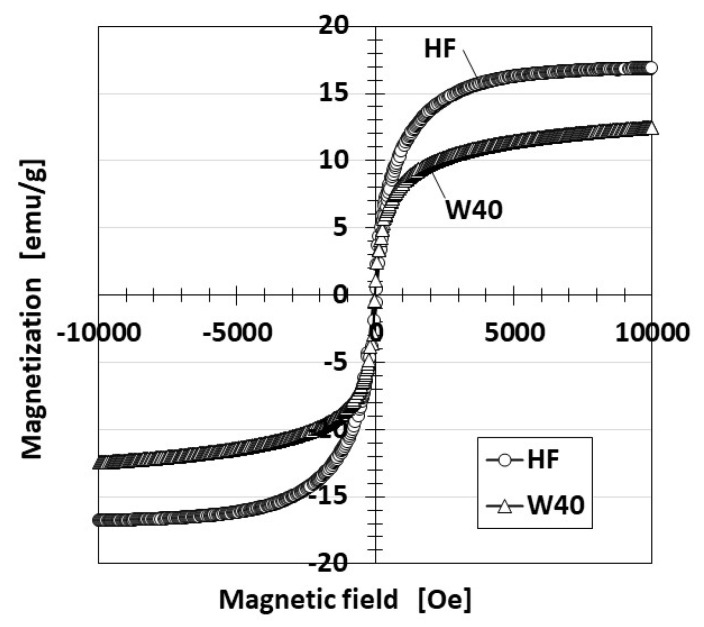
Magnetization of HF.

Figure 2 shows the magnetization of HF compared to MF measured by a vibrating sample magnetometer (VSM, TM-VSM1015-CRO-T, Tamakawa Co. Ltd., Sendai, Japan). The saturation magnetization of the HF specimens is several times larger than that of MF. However, it is not larger than that of other magnetic responsive fluids; MRF, for example, is the same regarding viscosity.

HF is coordinate with MF because its viscosity and magnetization are equivalent to those of MF based on the results shown in Figure 1 and Figure 2.

When utilizing HF in rubber, it is important to estimate the piezoelectricity and piezoresistivity of the electrolytically polymerized rubber. By another comparison of the piezoelectricity of electrolytically polymerized HF rubber using M300 and Ni (µm-order and pimple-like shapes on the surface; No. 123; Yamaishi), the spontaneous voltage using Ni is larger than that of M300. Detailed results of behavior, viscosity, magnetization of HF, as well as the optimal material in piezoelectricity and piezoelectricity are presented in another paper [27]. In addition, we adopted the utilization of Ni, as shown in the following section on the production of HF rubber sensors. Conveniently, the use of an outer power supply connected to a sensor in order to obtain an electrical signal from the sensor by the application of an electric field is dispensable. Because the use of an outer power supply is bulky and heavy, or the use of electric wires is obstructive for the operation of a robot. Therefore, the measurement of the spontaneous generating voltage in piezoelectricity is tractable on the operation.

## 3. Fabrication of Perceptible Rubber

### 3.1. Target Cutaneous Receptor and Skin

The above results demonstrate that HF is as useful as MCF in applications involving electrolytically polymerized rubber. It is also possible to use HF in the production of perceptible rubber.

**Figure A1:**
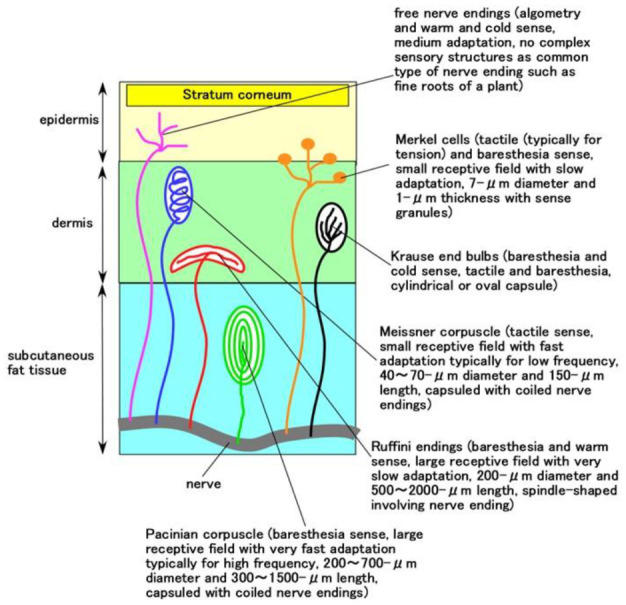
Schematic cutaneous structure and receptor in dermatology.

The human skin has five types of touch and temperature sensation that can be perceived by cutaneous receptors: tactile, baresthesia, algometry, warm, and cold [28] (see Figure A1 in the Appendix A). In dermatology, receptors such as Merkel cells, Meissner corpuscles, etc., are currently categorized as mechanoreceptors, thermoreceptors, and nociceptors. Because the object of the present study was to assess the viability of fabrication, we examined fabrication using HF rubber mimicking the structure of human skin involving cutaneous receptors.

In their morphology, free nerve endings have no complex sensory structures as in common types of nerve endings such as the fine roots of a plant (Figure A1) [29]. Therefore, in the present study, we simulated ramification with many thin electric wires. Krause endbulbs have a cylindrical or oval body containing the sheaths of many fibers and a core of soft semifluid (Figure A1). We simulated a capsule-like body embedded with many electric wires. Meissner corpuscles are encapsulated by many nerve endings surrounded by cells and tissues. The fibers meander between layers throughout the corpuscle (Figure A1) [30]. Therefore, we simulated this structure using coiled electric wires in a capsule-like body. Pacinian corpuscles are similar to Meissner corpuscles and have connective multi-layers [31]. Therefore, for our simulation, we created multi-layered rubber in a capsule-like body. Ruffini endings or Ruffini corpuscles have elongated capsules in a spindle shape involving branched nerve endings (Figure A1) [32]. Therefore, our simulation involved an elongated capsule-like body with many electric wires embedded in it.

We first fabricated the five types of the cutaneous receptor using HF rubber. Each cutaneous receptor has the schematic and organization of nerve endings shown in Figure A1: free nerve endings, Krause endbulbs, Meissner corpuscles, Pacinian corpuscles, Ruffini endings. We mimic the shape by replacing nerve endings with very thin electric wires and capsules with HF rubber. Next, the mimicked cutaneous receptor was embedded in U-rubber by molding and a body part was simulated. We adopt a thumb. U-rubber is highly stretchable, bendable, elastic enough to realize a soft motion of the body rather than any other rubber involving Q and NR.

### 3.2. Fabrication Procedure

#### 3.2.1. Preliminary of HF Rubber

**Figure 3:**
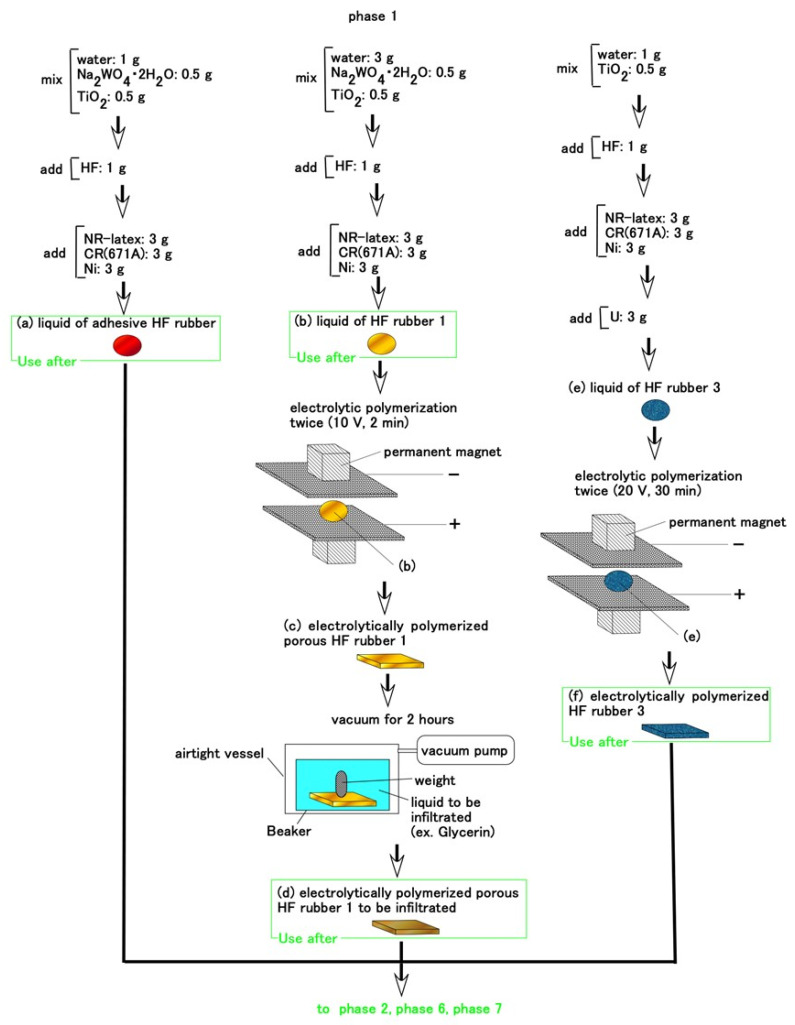
Preparation of an adhesive HF rubber liquid, a permeable solidified HF rubber and a husk solidified HF rubber in advance during Phase 1.

In advance, we prepared an adhesive HF rubber liquid, a permeable solidified HF rubber and a husk solidified HF rubber during Phase 1 (Figure 3). Throughout the production of HF rubber, the cause of compounding TiO_2_ is that it is electron transport material. Regarding adhesive HF rubber liquid, we used the adhesion technique for bonding between metal and rubber by electrolytic polymerization of diene rubber combined with a hydrate [33], demonstrating its effectiveness at bonding thin electric wire to a rubber. The hydrate, metallic hydrous oxide, sodium tungstate (VI) dehydrate, Na_2_WO_4_·2H_2_O (Fujifilm Wako Chemical Co., Ltd., Osaka, Japan), is compounded in NR-and CR-mixed latex. Where a-adhesive HF rubber liquid is so liquidity as to have a role of spreadable adhesive if more water such as 3 g is used. The liquid of adhesive HF rubber is used in the subsequent phases as an adhesive.

Regarding c-permeable solidified HF rubber 1, the rubber can be made porous by combining much water (which is a few times more than that of a-adhesive HF rubber liquid) at electrolytic polymerization using a hydrate, as described in our previous study. Then any liquids can percolate through the rubber so that the liquids can be permeated into the cavity [15]. In the present study, glycerin is permeated due to the spontaneous enhancing voltage of the HF rubber. When Na_2_WO_4_·2H_2_O is mixed into NR or CR immediately, the rubber solidifies. Therefore, Na_2_WO_4_·2H_2_O should be combined in advance, during the first stage, as shown in Figure 3. In addition, because TiO_2_ combines well, it is also combined with water in the first stage. When U (Human skin gel; Exseal Co., Ltd., Gihu, Japan) is combined with NR or CR immediately, they solidify. Therefore, U should also be added in advance, during the first stage, as shown in Figure 3. Because the rubber including U (e rubber liquid 3) has dwindling conductivity, both the voltage and the duration of the electrolytic polymerization should become larger than those of the rubber combined with only NR and CR. The intensity of our magnet was 180 mT and the electrode gap between the stainless plates during electrolytic polymerization was 1 mm.

#### 3.2.2. Fabrication of Cutaneous Receptors

Next, we produced five types of cutaneous receptors: free nerve endings, Krause and bulbs, Meissner corpuscles, Pacinian corpuscles and Ruffini endings.

**Figure 4:**
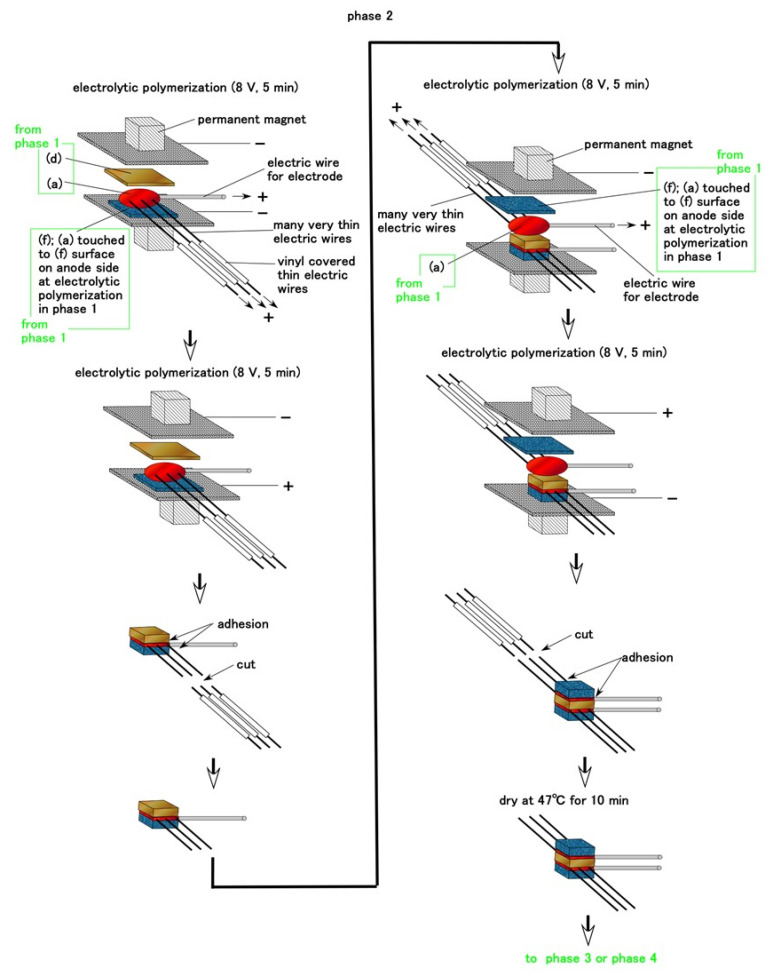
Schematic of the fabrication of cutaneous receptors of free nerve endings, Krause endbulbs and Meissner corpuscles during Phase 2.

We show the fabrication of free nerve endings, Krause endbulbs, and Meissner corpuscles during Phase 2, as shown in Figure 4. The very thin electric wires were approximately 0.1 mm in diameter and the electric wire for sensor electrodes had an outer diameter of approximately 0.8 mm with seven thin silver-gilt electric wires of roughly 0.1 mm in diameter inserted into adhesive rubber liquid a such that they were sandwiched between permeable rubber D and rubber f. Permeable rubber D is sandwiched between rubbers F by electrolytic polymerization using adhesive rubber liquid a. Here, attention must be paid to the side of rubber F exposed to adhesive rubber liquid a. The exposed side of rubber F facing outward should be the surface on the cathode side during electrolytic polymerization in Phase 1 and has the concave and convex shape shown in [34]. If the surface is the opposite, that is, the anode side during electrolytic polymerization, it will not be able to adhere well to U. Owing to its concave and convex shape, the surface that was on the cathode side can adhere to U. This technique is significant in the fabrication of artificial human skin. Spontaneous voltage occurs through the mediation of permeable rubber d.

**Figure 5:**
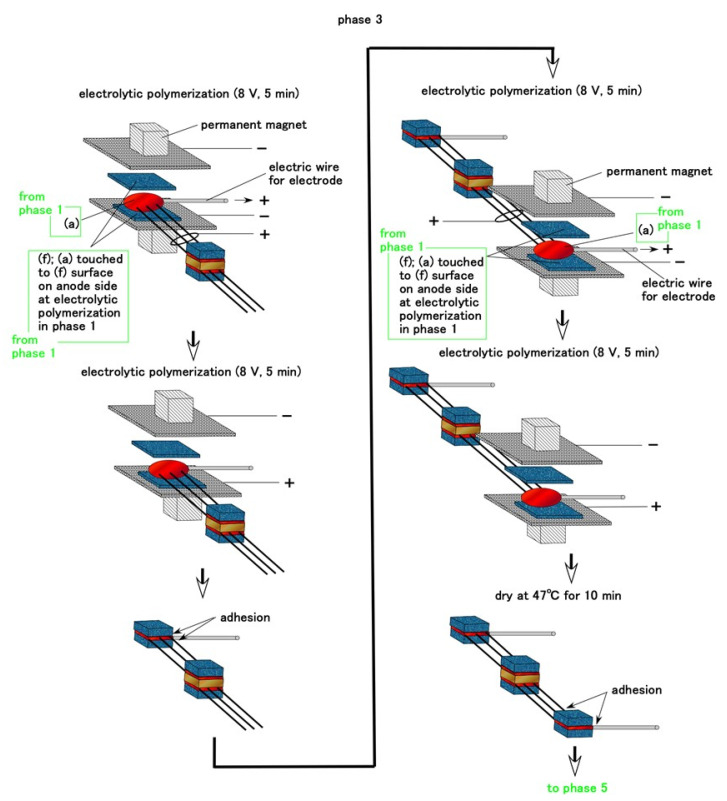
Schematic of the fabrication of Meissner corpuscle cutaneous receptors during Phase 3.

For Meissner corpuscles, many very thin electric wires are inserted between permeable rubber D and rubber F during Phase 2. During Phase 3, the electric wire for the sensor electrode is inserted between other rubbers f, as shown in Figure 5. Therefore, the current between the electrode wires flows through the many very thin electric wires and the electrolytically polymerized rubber with rubbers F and d.

**Figure 6:**
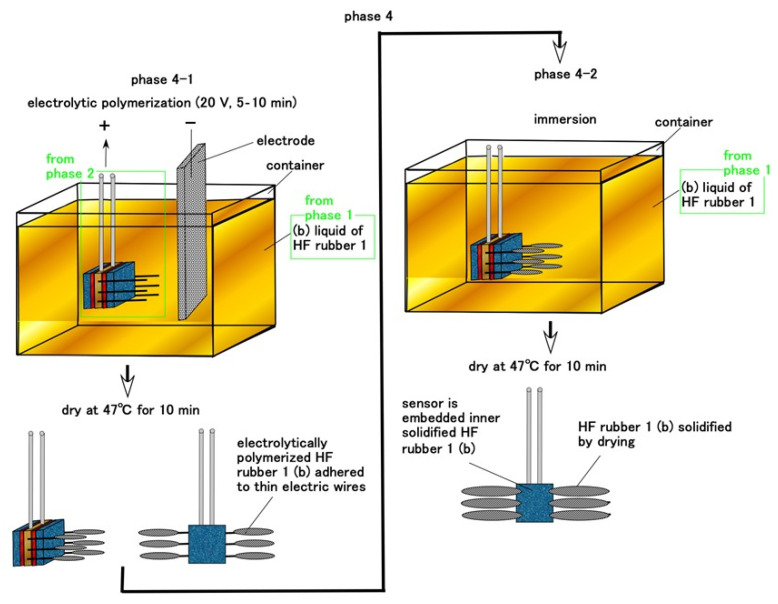
Schematic of the fabrication of free nerve endings and Krause endbulbs as cutaneous receptors during Phase 4.

Regarding free nerve endings and Krause endbulbs, the consummated HF sensor at Phase 2 is immersed in the B HF rubber liquid 1 in Phase 1 and electrolytically polymerized, as shown in Figure 6. The vulcanization of the rubber liquid begins at the anode and expands from the anode, as shown in our previous studies [34]. Therefore, the B HF rubber liquid 1 is vulcanized on the anode side of the sensor, and the vulcanized rubber liquid then adheres to the thin electric wires. However, at Phase 4-1, we can control the amount of the rubber vulcanized on the thin electric wires by the time during electrolytic polymerization. For example, the vulcanized rubber is tenuous when we provide 5 min such as shorter time, and it is thick when we provide 10 min. The direction of the many branches of thin electric wires can be changed freely by bending.

**Figure 7:**
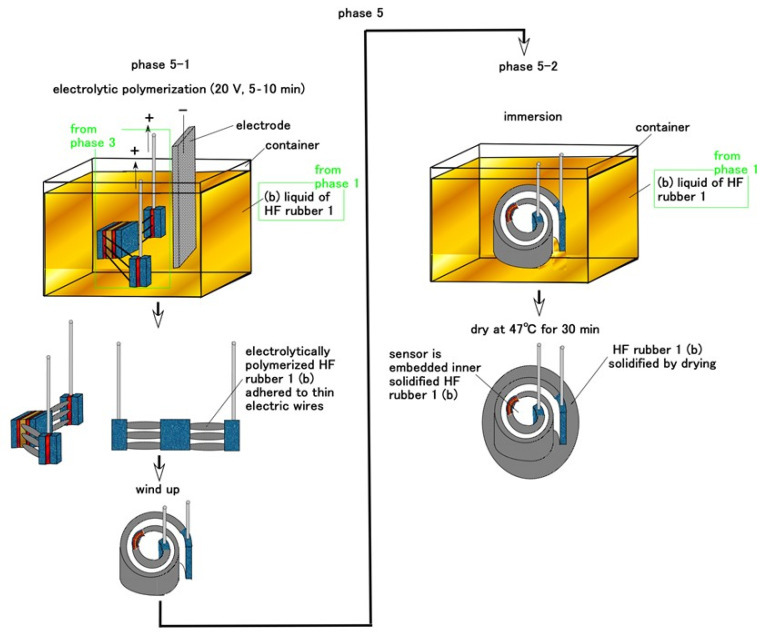
Schematic of the fabrication of Meissner corpuscle cutaneous receptors during Phase 5.

For Meissner corpuscles, the HF sensor consummated during Phase 3 is embedded in the inner part of B HF rubber liquid 1 in Phase 1 and electrolytically polymerized during Phase 5, as shown in Figure 7. Such as in Figure 6, the many thin electric wires are attached by vulcanized HF rubber liquid b. The coiled HF rubber sensor is then embedded in B HF rubber liquid 1 in Phase 1 again and dried; B HF rubber liquid 1 solidifies around the coiled HF rubber sensor.

**Figure 8:**
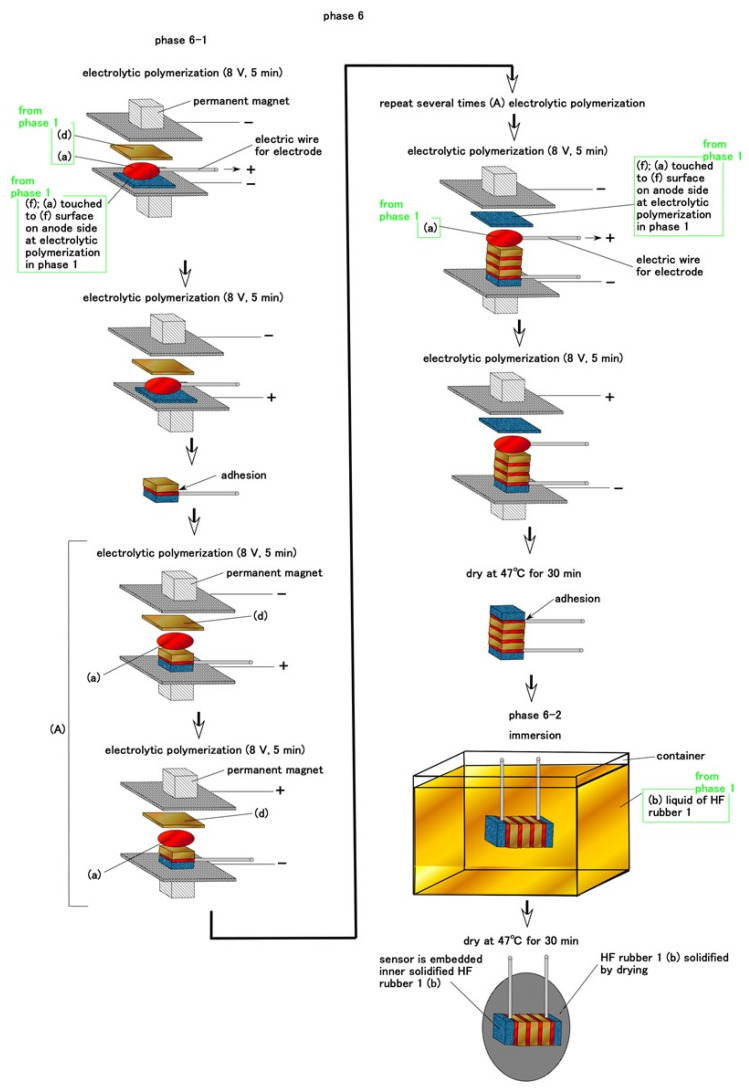
Schematic of the fabrication of Pacinian corpuscle cutaneous receptors during Phase 6.

Regarding Pacinian corpuscles, the electrolytic polymerization is repeated without inserting thin electric wires during Phase 6 as shown in Figure 8. The HF rubber sensor is embedded in B HF rubber liquid 1 in Phase 1 and dried. The rubber liquid then solidifies around the HF rubber sensor.

**Figure 9:**
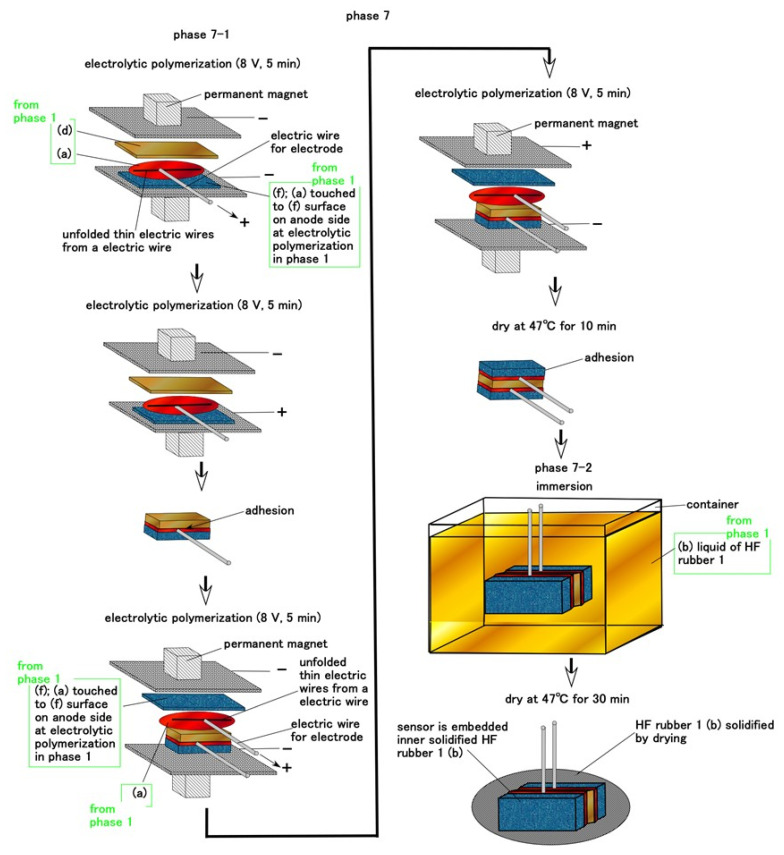
Schematic of the fabrication of Ruffini ending cutaneous receptors during Phase 7.

In the case of Ruffini endings, the electrolytic polymerization of the sandwiched layers between permeable rubber D and rubber F is carried out without inserting thin electric wires during Phase 7, as shown in Figure 9. The HF rubber sensor is embedded inB HF rubber liquid 1 in Phase 1 and dried. The HF rubber liquid then solidifies around the HF rubber sensor.

The ultimately fabricated HF sensor in Phases 4-2, 5-2, 6-2, 7-2 is a prerequisite to achieve thorough aridity because of the solidification of U as shown in the following procedure. If the HF sensor is insufficiently dry, U cannot solidify well.

**Figure 10:**
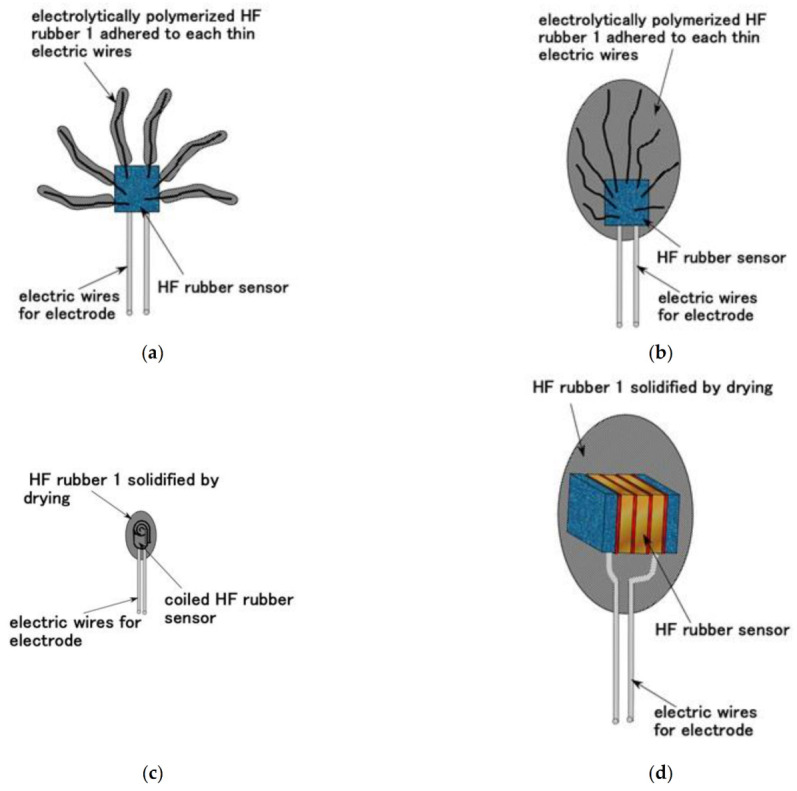

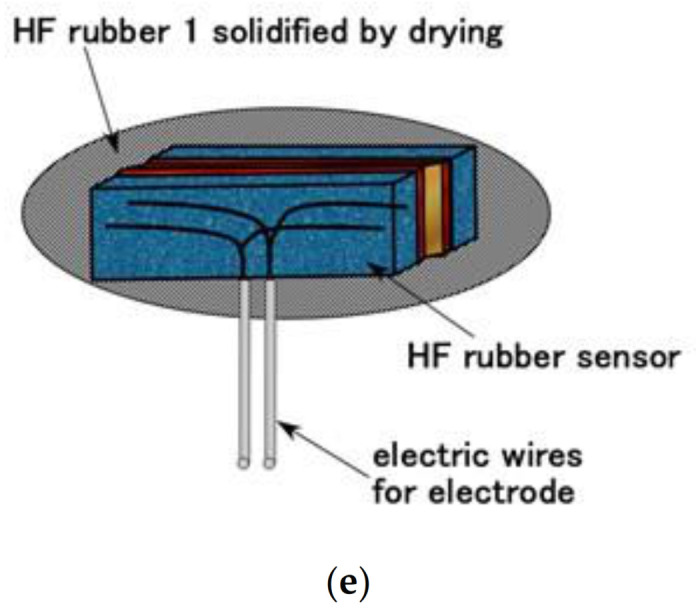
Consummated fabric of each cutaneous receptor mimicking human skin: (**a**) Type-A HF sensor for free nerve endings; (**b**) Type-B HF sensor for Krause and bulbs; (**c**) Type-C HF sensor for Meissner corpuscles; (**d**) Type-D HF sensor for Pacinian corpuscles; (**e**) Type-E HF sensor for Ruffini endings.

**Figure 11:**
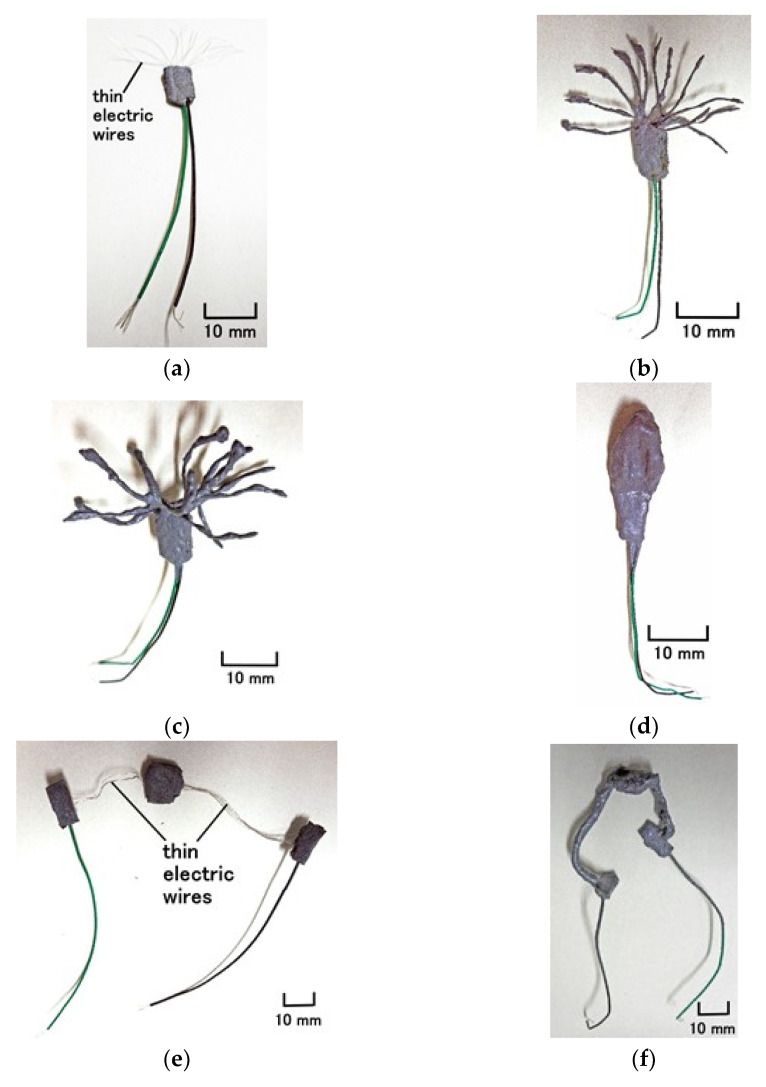

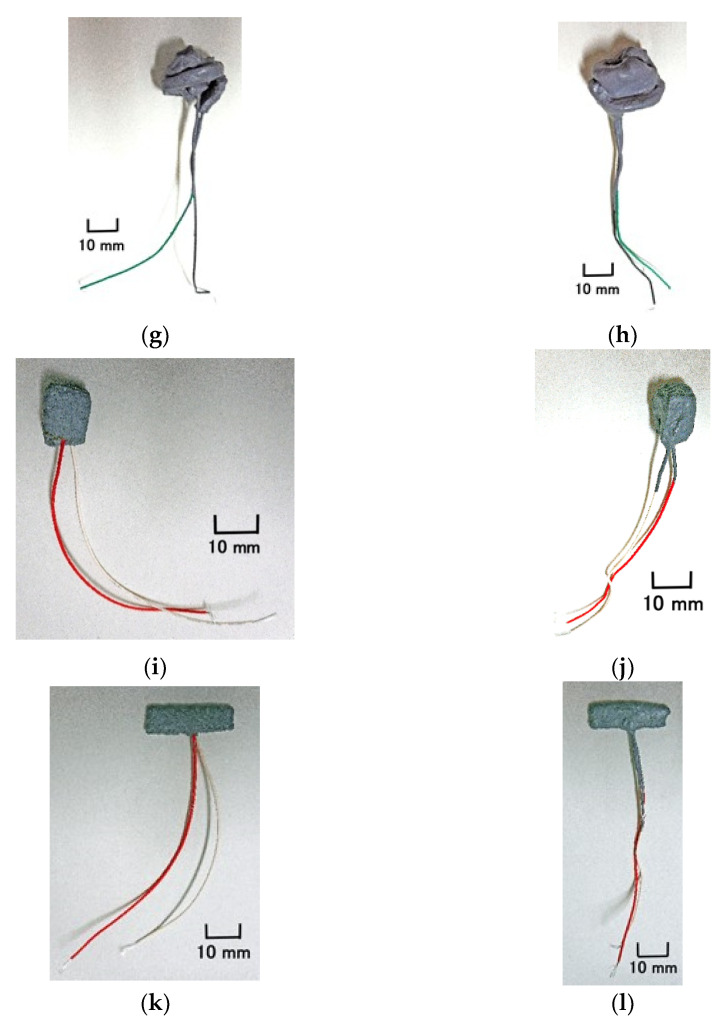
Photographs of the consummated fabric of each cutaneous receptor produced by electrolytic polymerization with HF: (**a**) consummated fabric at Phase 2 in Figure 4; (**b**) consummated fabric at Phase 4-1 in Figure 6; (**c**) consummated fabric at Phase 4-2 in Figure 6; (**d**) consummated fabric of (**b**) to be bundled, immersed and dried during Phase 4-2 in Figure 6; (**e**) consummated fabric at Phase 3 in Figure 5; (**f**) fabric electrolytically polymerized during Phase 5-1 in Figure 7; (**g**) consummated fabric to be coiled during Phase 5-1 in Figure 7; (**h**) consummated fabric at Phase 5-2 in Figure 7; (**i**) consummated fabric at Phase 6-1 in Figure 8; (**j**) consummated fabric at Phase 6-2 in Figure 8; (**k**) consummated fabric at Phase 7-1 in Figure 9; (**l**) consummated fabric at Phase 7-2 in Figure 9.

As a result, regarding free nerve endings, the fabric of a Type-A HF sensor has many branches adhered to HF rubber (Figure 6 and Figure 10a). Regarding Krause and bulbs, the fabric of a Type-B HF sensor is integrated HF rubber embedded with many electric wires, as shown in Figure 10b. This fabric can be obtained through long electrolytic polymerization during Phase 4 (Figure 6). Regarding Meissner corpuscles, the fabric of a Type-C HF sensor is the consummated HF sensor at Phase 5, as shown in Figure 7 and Figure 10c. Concerning Pacinian corpuscles, the fabric of a Type-D HF sensor is the consummated HF sensor at Phase 6, as shown in Figure 8 and Figure 10d. With respect to Ruffini endings, the fabric of a Type-E HF sensor is the consummated HF sensor at Phase 7, as shown in Figure 9 and Figure 10e. The mimicking cutaneous receptors shown in Figure 10 are produced as shown in Figure 11.

#### 3.2.3. Human Body Embedded Cutaneous Receptors

**Figure 12:**
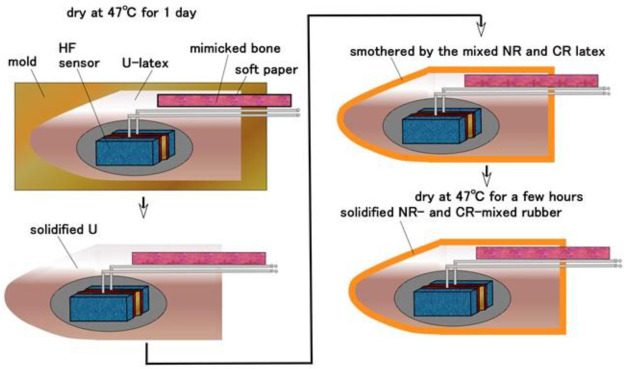
Fabrication of a finger with each of the cutaneous receptors embedded in U-rubber.

**Figure 13:**
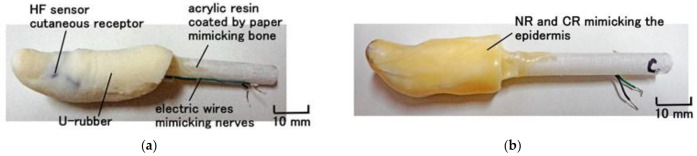
Photographs of a finger with each of the cutaneous receptors: (**a**) before coating with mixed NR and CR; (**b**) after coating with mixed NR and CR.

Next, we fabricated a human thumb embedded with each type of cutaneous receptor in U rubber (Figure 12). In the present paper, we primarily adopted a thumb in many human body parts so that we embedded each fabricated receptor in each thumb because we investigated each sensitivity of five fabricated receptors. First, the fabricated receptor immersed in U-latex (Figure 10 and Figure 11) in a finger-shaped molding is dried. Because the exposed side of rubber F facing outward is the surface that was on the cathode side during electrolytic polymerization in Phase 1, the surface of F can adhere to U, as mentioned above. Because the finger was caught on a measuring instrument during installation, we covered the rigid body having 8 mm in diameter with a soft paper such as tissue paper which is adhered by double-coated adhesive tape and inserted inner the U-latex. The rigid body is made of acrylic resin and mimicked human bone. A soft paper was used to ensure good adhesion between the paper and U at solidification. Care must be taken to ensure that the embedded cutaneous receptor is dried well before U solidifies. Next, the solidified-U body is coated by the mixed NR and CR latex whose weight ratio is 1:1, and dried as shown in Figure 13. U corresponds to subcutaneous fat tissue and the solidified NR- and CR-mixed rubber to the epidermis or dermis. It is important not to coat with only NR, because NR cannot adhere to the solidified U surface; the blend of NR and CR is therefore essential. If the mimicked human body is to be used in harsh environmental conditions, it should be coated with Q. Therefore, HF rubber 3 liquid (Figure 3) is coated on the solidified NR and CR-mixed rubber to be dried for a few hours, and then Q-latex is coated on the solidified 3-HF rubber to be dried for a few hours again. HF rubber 3 is used to achieve the adhesion of Q on the solidified NR- and CR-mixed rubber. The electrode wires from the HF sensor correspond to nerves.

## 4. Mechanical and Thermal Sensing

We investigated the characteristics of the produced thumb with HF sensors mimicking cutaneous receptors by using a simple experimental apparatus. For the way to estimate the sensibility of a mimetic organ of perceptual cells in a human body, we adopted the ordinary measurement of voltage from the fabricated organ for the application of any forcing and heating. Because the present fabricate is a novel haptic sensor, the predefined parameters estimated the electrical signal from the novel haptic sensor had not been stipulated. Until recently, there have not been studies on the fabrication of a mimetic organ of perceptual cells in a human body. The measurement of the data, such as the voltage for the application of any forcing and heating, is the latest issue in the field of fusion of engineering and medical science so that it might be developed for the future. On the other hand, the key issue of the paper is the preliminary work of trying the fabrication of the mimic organ. Therefore, in the present paper, at the primary level of the measurement, the voltage for the application of any forcing and heating is employed.

**Figure 14:**
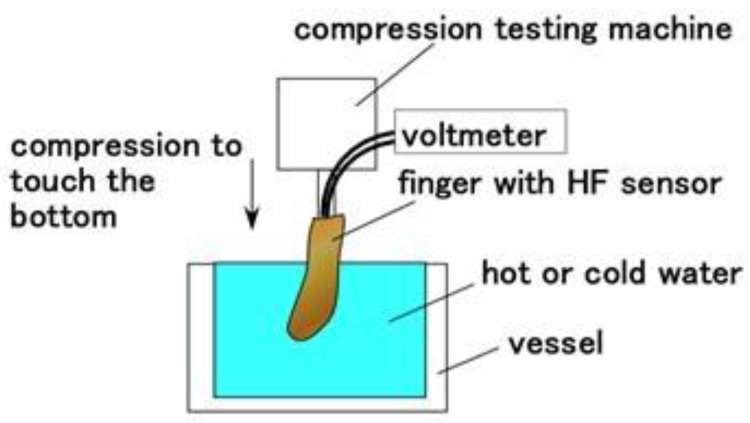
Schematic diagram of the experimental apparatus of touching thermal water and bottom of a vessel.

**Figure 15:**
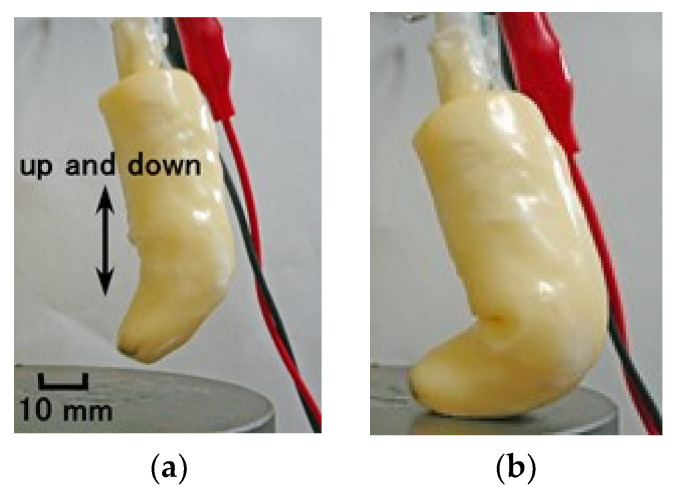
Photographs of the soft bending finger: (**a**) before touching; (**b**) upon touching.

At first, as shown in Figure 14, we investigated the response to the application of normal force and thermal body by using the same normal force experiment (NFE) apparatus [34]. The finger was moved to touch hot or cold water, continuing to touch the bottom of the vessel. The up and down motion in the water was repeated five times by a compression testing machine (SL-6002; IMADA-SS Co., Ltd., Toyohasi, Japan) at a velocity of 300-mm/min. The voltage from the sensor was measured by a voltage meter (PC710, Sanwa Electric Instrument Co. Ltd., Tokyo, Japan). The finger can bend very softly, as shown in Figure 15.

**Figure 16:**
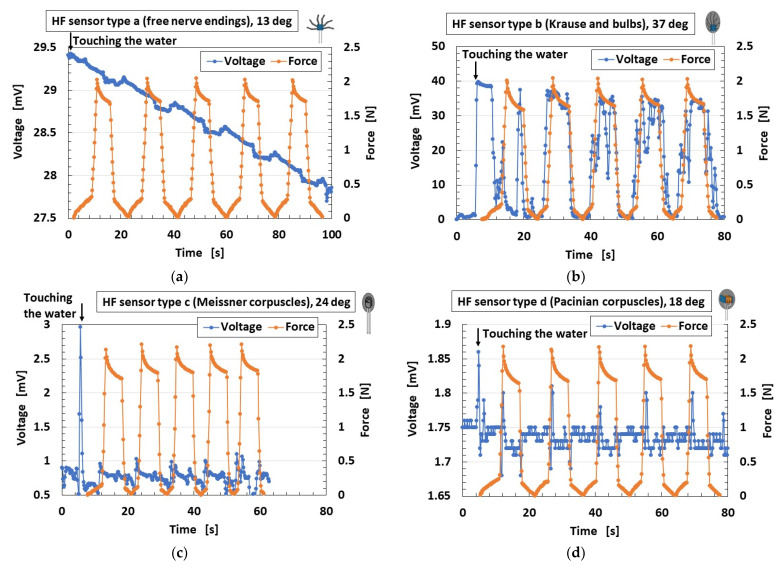

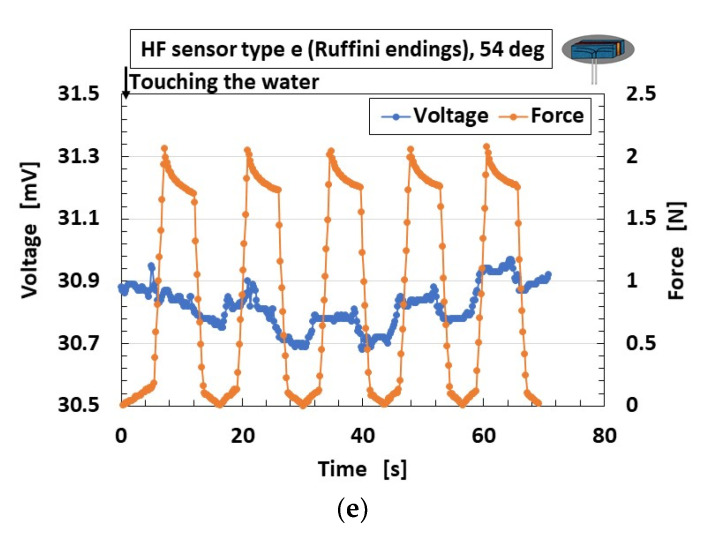
Voltage of each of the cutaneous receptors at compression: (**a**) Type A; (**b**) Type B; (**c**) Type C; (**d**) Type D; (**e**) Type E.

Figure 16 shows the results at each HF sensor cutaneous receptors (Types A–E). The instant that the finger touches the water is indicated in each case. Regarding thermal response, Figure 16b–d show that the HF sensor is sharply responsive to the thermal input. In the case of the Type-B sensor, the thermal response is conspicuous. There is no thermal response in Types A and E. This sharp response in a human is so effective that the human can perceive the thermal input instantly upon touching with shouting. Therefore, the goal of the present simulation is the perception of a healthy human. The fabrication of the HF sensor, both in its materials and in its multi-layer structure, contributes to its ability to receive thermal input.

Regarding the response to force, the change in voltage in Type-B is larger than that of any of the other types. An instantaneous decrease in voltage appears occasionally during the application of compression. This is due to the behavior of the ions in the HF rubber: the voltage increases with decreasing distance between ions; however, the voltage becomes zero when the ions reach contact. The same tendency has been confirmed in MCF rubber [35]. The change in voltage in Types C and D is small, however, and essentially remains constant. In contrast, Types A and E are very small and increase or decrease as a slow wave. We hypothesize that the fabrication of the HF sensor with many electric wires as in Type B, or with multi-layer structures as in Types B, C and D, contributes to the ability of the sensor to receive input about force.

**Figure 17:**
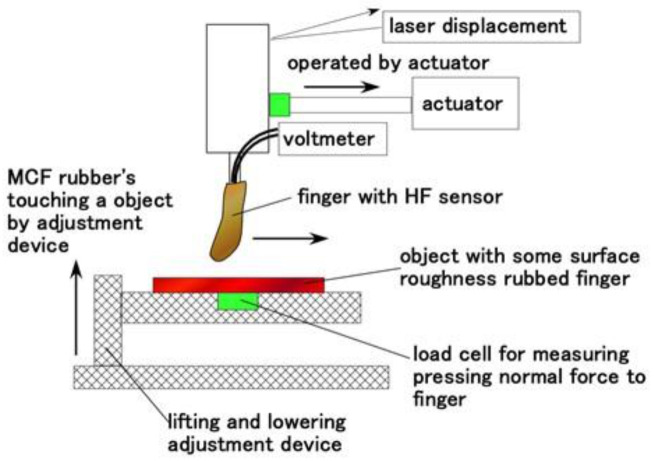
Schematic diagram of the experimental apparatus of stroking a body with some surface roughness.

Next, as shown in Figure 17, we investigated the response to the application of shear force by using the same shear force experiment (SFE) apparatus [34]. The finger came into contact with a rubbed object and installed on an actuator with 50-mm/min sweeping speed, 50-mm sweeping distance and around 0.02-N normal force, was swept parallel to the object with some surface roughness by adjusting a constant height interval by adjustment device. The object is of two types: alternate surface roughness using a few sandpapers and a body concave and convex. The normal force on the finger was measured by a load cell, and the total distance by a laser displacement.

**Figure 18:**
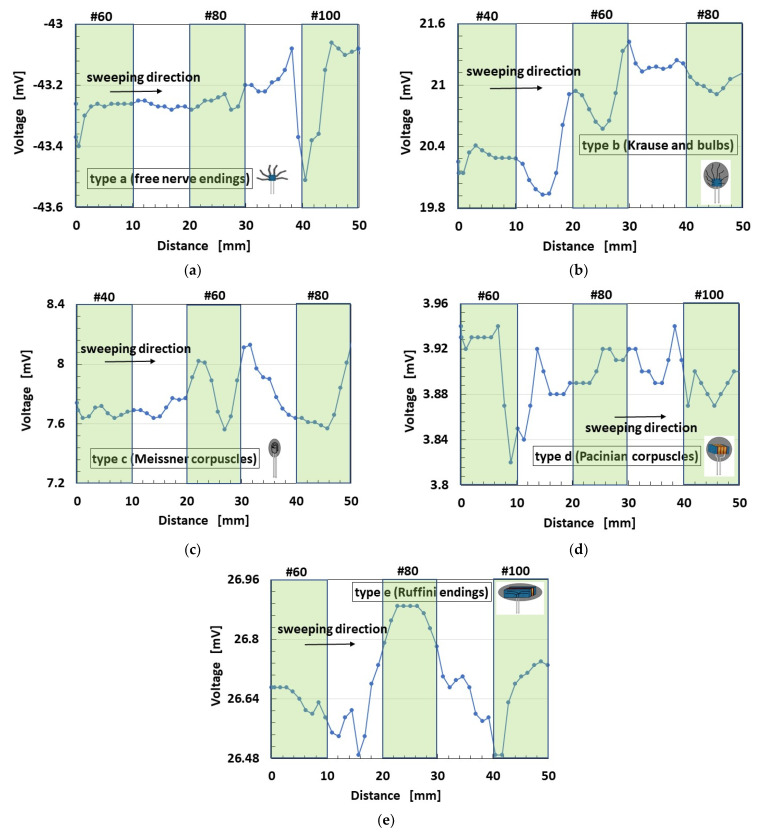
Voltage of each of the cutaneous receptors at shear motion on a body with alternate surface roughness: (**a**) Type A; (**b**) Type B; (**c**) Type C; (**d**) Type D; (**e**) Type E.

Figure 18 shows the spontaneous voltage by stroking alternate sandpapers with a surface roughness of #40 (the arithmetic average height *R_a_* = 41.11 μm, root mean square height *R_q_* = 48.91 μm, the difference of height between maximum and minimum *R_y_* = 170.3 μm), #60 (*R_a_* = 30.91 μm, *R_q_* = 36.38 μm, *R_y_* = 139.4 μm), #80 (*R_a_* = 24.11 μm, *R_q_* = 28.6 μm, *R_y_* = 103.9 μm) sandwiched by a sleek acrylic resin surface between them (*R_a_* = 0.03 μm, *R_q_* = 0.03 μm, *R_y_* = 0.2 μm). The surface roughness was measured by a surface roughness measuring device (SJ-400, Mitutoyo, Co. Ltd., Kawasaki, Japan). The white areas of Figure 18 are the one of acrylic resin. The figure also indicates that the sweeping direction is from the left to right side, shown by arrow.

The changes in voltage in Types B and C are larger than any other types. However, Type E is the most responsive to the surface roughness of sandpapers, although the change in voltage is small. The cause is guessed that type E can be responsive to a shear motion because the lateral length of sensor is long.

**Figure 19:**
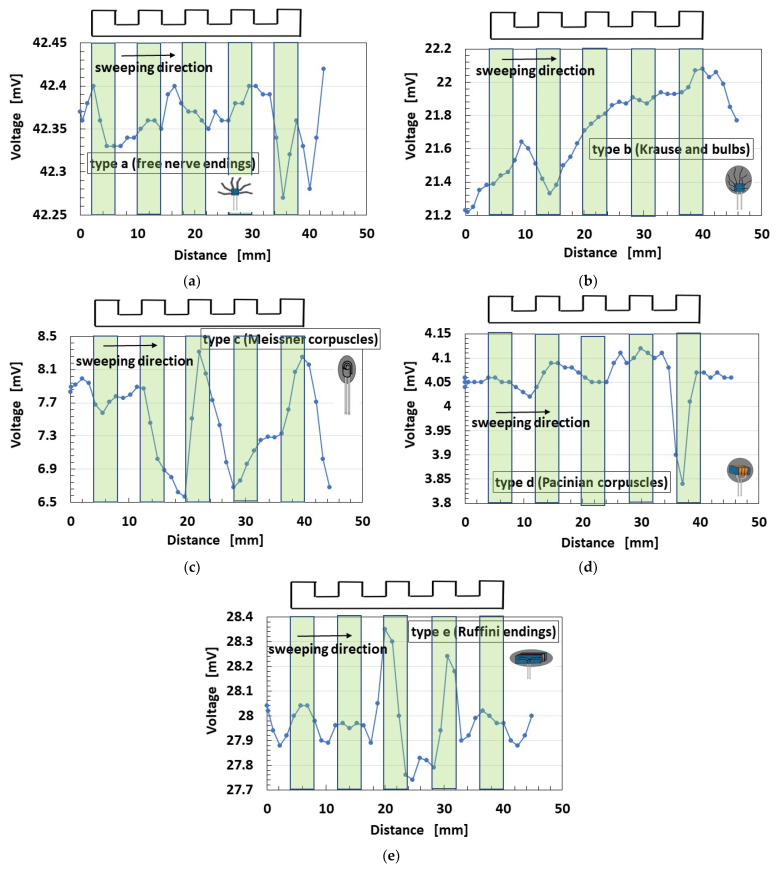
Voltage of each of the cutaneous receptors at shear motion on a concave and convex body: (**a**) Type A; (**b**) Type B; (**c**) Type C; (**d**) Type D; (**e**) Type E.

**Figure 20:**
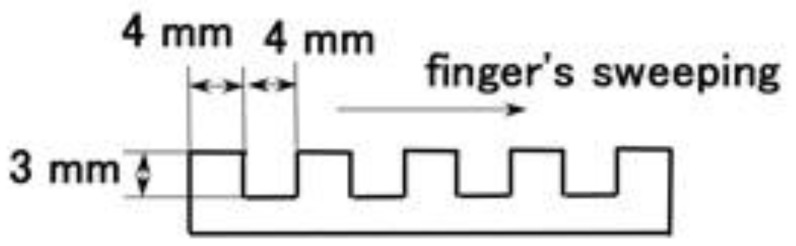
Schematic diagram of the concave and convex body in stroking experiment.

Figure 19 shows the spontaneous voltage by stroking a concave and convex body. The detail dimension is shown in Figure 20. The figure indicates that the position of the concave and convex body corresponds to the change in voltage and that the sweeping direction is from left to right side shown by arrow.

As shown in Figure 18, the changes in voltage in Types B and C are larger than any other type. However, Type E is the most responsive to the surface roughness of convex parts, although the change in voltage is small. This denotes that type E can be responsive to a shear motion because the lateral length of sensor is long.

The measured data of voltage have the accuracy of 0.001 mV by our used instrument. Therefore, the change in the voltage has significant difference. The measured data of voltage could be obtained quantitatively and qualitatively repeatedly. Based on the accuracy, our fabricated mimicked perceptual cells present three peculiarities: the change of the voltage by forcing or seeping the groove is large, as shown in Figure 16b and Figure 18a–c, except for thermo-sensibility; the change of the voltage by them is small and the voltage changes around a large value, as shown in Figure 16a,e, Figure 18e and Figure 19a,b,e; the change of the voltage by them is small and the voltage changes around a large small value, as shown in Figure 16c,d, Figure 18d and Figure 19c,d, except for thermo-sensibility. The cause of these three peculiarities is due to the structured style of the fabricate.

Our experimental results are not completely similar to previous results in the dermatology field on cutaneous receptors. The cause of this can be could be due to the fact that there is room for improvement on the fabrication and the location of cutaneous receptors contrary to our proposed fabrication and arrangement in the present study. In addition, the effect of the size of the fabricated cutaneous receptors could be due to their very small size, or the interactions of the complicated systems among cutaneous receptors under the operation of plural variegated cutaneous receptors, etc. However, in the present study, the purpose of the proposition of the fabrication of cutaneous receptors embedded in a body for robotics was achieved. The experimental data presented here is an example of the results obtained under various experimental conditions, such as response to vibration, electromagnetic waves, etc. Further results obtained under other experimental conditions will be addressed in other reports.

## 5. Conclusions

Our novel magnetic responsive fluid, HF, is so useful in the production of conductive soft rubber using electrolytic polymerization that it can be mixed with both diene and non-diene rubbers. HF consists of a combination of water, kerosene, KF96 and PVA. The viscosity and the magnetization of HF are equivalent to those of other magnetic responsive fluids, MF. For the production of the rubber sensor by the combination of HF and rubber, the piezoelectric characteristics of compounded rubber with HF indicate that the Ni compound is optimal. The cause is the convenience that using an external power supply and electric wires is dispensable for the operation of the robot installed a haptic sensor made of HF rubber.

By utilizing our previously proposed novel techniques for rubber vulcanization and adhesion between rubber and metal by electrolytic polymerization, we addressed five cutaneous receptors: free nerve endings, Krause and bulbs, Meissner corpuscles, Pacinian corpuscles and Ruffini endings. In addition, we proposed a fabrication technique for a thumb with HF rubber sensors embedded in U-rubber. The technique of adhesion of electric wires onto the HF rubber sensor and the rubber was utilized and effective to avoid electric wires’ desorption from the sensor by large elongation or deformation of the body. In an experiment involving the finger touching cold or hot water to test sensing by mechanoreceptors or thermoreceptors in the mimicked human tissue, we found that the spontaneous voltage generated from the finger for mechanical and thermal sensing depends on its fabric. For thermal sensibility, the Type-B HF sensor mimicked Krause and bulbs, the Type-C HF sensor mimicked Meissner corpuscles, and the Type-D HF sensor mimicked Pacinian corpuscles type have the most thermal responsive in any other cutaneous receptors: 10-mV ordered large changing voltage for Type B, 1-mV ordered changing voltage for Type C, 0.1-mV ordered changing voltage for Type D. For pressing sensibility, the Type-B HF sensor mimicked Krause and bulbs is the most responsive to the pressure with 10-mV ordered large changing voltage. The other cutaneous receptors have responsive to the pressure with 0.1-mV ordered small changing voltage. For shearing sensibility, the Type-E HF sensor mimicked Ruffini endings is the most responsive to the surface roughness with 0.1-mV ordered small changing voltage in any other cutaneous receptors.

There are points that do not align with previous results known in the dermatology field on cutaneous receptors. This situation is not typical just for the present study. In general, many current varied mimicked materials in soft robotics do not yet reach the actual biological phenomena. Therefore, we must continue to investigate and elucidate the phenomena and simulate the actual biological phenomena. In addition, the response time of the voltage created from our fabricated sensor is also such a significant issue that it should be investigated. However, based on the results presented here, the fabrication of a finger involving simulated cutaneous receptors appears viable, though further experimental investigation is needed for more detail. The technique used in the present fabrication is suitable and helpful for the production of artificial cutaneous receptors embedded in artificial rubber skin mimicking human skin and installed in a humanoid or human-like robot in the future.

## Data Availability

Not applicable.

## Appendix A

Figure A1 shows the cutaneous structure and receptors. This schematic diagram is an organizational chart of human tissue used in dermatology.
